# The Alteration of Intestinal Microbiota Profile and Immune Response in *Epinephelus coioides* during Pathogen Infection

**DOI:** 10.3390/life11020099

**Published:** 2021-01-28

**Authors:** Joan Tang Xiao Joe, Yung-Che Tseng, Jen-Leih Wu, Ming-Wei Lu

**Affiliations:** 1 Marine Biotechnology, National Taiwan Ocean University, Keelung 20224, Taiwan; joan94_0137@live.cn; 2 Marine Biotechnology, Academia Sinica, Taipei 11529, Taiwan; 3 Marine Research Station, Institute of Cellular and Organismic Biology, Academia Sinica, Taipei 11529, Taiwan; yctseng@gate.sinica.edu.tw; 4 Institute of Cellular and Organismic Biology, Academia Sinica, Taipei 11529, Taiwan; jlwu@gate.sinica.edu.tw; 5 Department of Aquaculture, National Taiwan Ocean University, Keelung 20224, Taiwan; 6 Center of Excellence for the Oceans, National Taiwan Ocean University, Keelung 20224, Taiwan

**Keywords:** intestinal microbiota, *Epinephelus coioides*, nervous necrosis virus, grouper iridovirus, *Vibrio harveyi*, immune response

## Abstract

*Epinephelus coioides*, or grouper, is a high economic value fish species that plays an important role in the aquaculture industry in Asia. However, both viral and bacterial diseases have threatened grouper for many years, especially nervous necrosis virus, grouper iridovirus and *Vibrio harveyi*, which have caused a bottleneck in the grouper industry. Currently, intestinal microbiota can provide novel insights into the pathogenesis-related factors involved in pathogen infection. Hence, we investigated the comparison of intestinal microbiota communities in control group and pathogen-infected grouper through high-throughput sequencing of the 16S rRNA gene. Our results showed that microbial diversity was decreased, whereas microbial richness was increased during pathogen infection. The individuals in each group were distributed distinctly on the PLSDA diagram, especially the GIV group. *Proteobacteria* and *Firmicutes* were the most abundant bacterial phyla in all groups. Interestingly, beneficial genera, *Faecalibacterium* and *Bifidobacterium*, predominated in the intestines of the control group. In contrast, the intestines of pathogen-infected grouper had higher levels of harmful genera such as *Sphingomonas*, *Atopostipes*, *Staphylococcus* and *Acinetobacter.* Additionally, we investigated the expression levels of innate and adaptive immune-related genes after viral and bacterial infection. The results revealed that immunoglobulin T and proinflammatory cytokine levels in the intestine increased after pathogen infection. Through these unique bacterial compositions in diseased and uninfected fish, we could establish a novel therapeutic approach and bacterial marker for preventing and controlling these diseases.

## 1. Introduction

Orange-spotted grouper (*E. coioides*) is an important species with high economic value in Asia. Owing to the fast growing and spawning of grouper year-round at temperatures from 22 °C to 28 °C, the orange-spotted grouper is an easier species to culture compared to other grouper species. Unfortunately, the grouper has suffered from the spread of infectious diseases, which have caused mass mortalities, in recent years. Nervous necrosis virus (NNV), grouper iridovirus (GIV) and *V. harveyi* were reported as the leading causes of death in the early life cycle of grouper [1,2,3].

The main cause of disease in cultured grouper is viral pathogens including NNV and GIV. NNV is one of the most threatening viral diseases affecting over 120 species of cultured marine fishes worldwide, especially post-hatch larvae of *Epinephelus* spp., and the mortality ranges from 90 to 100% [1,4,5]. NNV mainly attacks the central nervous system and is associated with viral encephalopathy and retinopathy (VER), vacuolation in the brain and eyes, abnormal swimming, dark body color and even anorexia [4,6]. Other major pathogens, such as GIV of the genus *Ranavirus*, also cause severe mortalities of up to 60% and lead to huge economic loss in the grouper industry [7,8]. The symptoms of infectious grouper include darker skin color, and the gills may bleed and become congested; additionally, the target organs (spleen and head kidney) become swollen [3,9]. Furthermore, the family *Vibrionaceae* is most often associated with bacterial infection in grouper [10]. *V. harveyi* has also been referred to as opportunistic bacteria in *Lates calcarifer* [11], *Lateolabrax japonicus* [12] and *Litopenaeus vannamei* [13]. According to previous studies, *V. harveyi* destroys the tissues on the epidermis and cause skin ulceration called skin ulcer disease [10].

Recently, many studies have revealed that diseases influence the intestinal microbiota composition [14], not only in mammals but also in fish species. The intestine is a complex organ composed of beneficial and harmful bacteria; thus, a beneficial intestinal microbiota has been highlighted as having a key role in maintaining proper digestive functioning [15,16]. The disruption of intestinal microbiota has been reported to correspond with fish after pathogen infections, including infections in *Salmo salar*, *Oncorhynchus mykiss*, *Larimichthys crocea* and *Hippocampus kuda* [17,18,19,20]. As mentioned above, a series of studies have stated that the composition of intestinal flora is altered by pathogen infection; however, the actual mechanism is still unknown. Therefore, the interaction of pathogens and intestinal microbiota is becoming an attractive topic for microbiological and medical research [21].

We previously developed an intestinal microbiota database of healthy grouper throughout metamorphosis stages. This database revealed that distinct bacterial composition dominated in each stage [22]. To enrich the knowledge about the alteration in intestinal microbiota upon viral and bacterial pathogen infection, we focused on comparing microbial communities of control group with NNV, GIV-and *V. harveyi*-infected grouper in this study. In addition, we evaluated the mRNA gene expression levels of cytokines-mediated inflammatory factors and immunoglobulins in grouper intestine after viral and bacterial infection. We expected that the intestinal microbiota profile could help us understand the interaction between the host and intestinal microbiomes. Through these data, we established a novel therapeutic approach and bacterial marker for preventing and controlling these diseases.

## 2. Materials and Methods

### 2.1. Sampling and Challenge of Juvenile Grouper

In this study, juvenile of orange-spotted groupers were collected from hatcheries at Pingtung, Taiwan. The animal experiment was conducted at National Taiwan Ocean University (Keelung, Taiwan) by following the institutional IACUC guideline (approval number: 106009). The juveniles (1–2 cm) were daily fed a mixture of commercial feeds and Artemia nauplii (0.2 g). NNV and GIV titers were determined using TCID_50_ (50% tissue culture infective dose) in grouper fin cells (GF-1) and grouper kidney cells (GK) [23]; the LD_50_ test of *V. harveyi* was performed according to Li J et al., 2019 [24]. The healthy groupers were separated into four groups in duplicate: the control (n = 20), NNV (n = 20), GIV (n = 20) and Vibrio (n = 20) groups. Fish were acclimatized in 5L tanks equipped with a storage tank system at 30 ℃ with a salinity of 32‰ and constant aeration. The juveniles were injected intraperitoneally with NNV (10^6.6^ TCID_50_/mL), GIV (10^8^ TCID_50_/mL) and *V. harveyi* (6.4 × 10^7^ CFU/mL). The sample collection procedures were performed according to Wayne Knibb et al., 2017 [25]. We collected the intestine samples from five individuals in each tank. All the pathogen infected grouper displayed obvious clinical signs with abnormal swimming behavior (NNV), lethargy (GIV), lesions on skin and tail (Vibrio) with the mortality occurring after seven and 14 days post infection (dpi) with virus and bacteria, respectively. No clinical signs were observed in the control groups. Furthermore, the affected tissues (NNV: brain; GIV: head kidney; *V. harveyi:* liver) were homogenized and the viruses and bacteria detected by PCR using specific primers for NNV, GIV or *V. harveyi*. In addition, the intestine and head kidney samples of all groups were collected for RNA and DNA extraction. After dissection, intestine and head kidney samples were washed twice with PBS and immediately stored at −80 ℃ until subsequent use. 

### 2.2. RNA Extraction and cDNA Synthesis

Total RNA was extracted from intestine and head kidney samples by using TRIzol^®^ Reagent (Invitrogen, Carlsbad, CA, USA) following the manufacturer’s instructions, and DNase I treatment (Thermo Scientific™, Waltham, MA, USA) according to the manufacturer’s instructions. The quality of extracted RNA was determined on a 1% agarose gel by electrophoresis. After that, total RNA (1μg) was used for cDNA synthesis by using HiScript I Reverse Transcriptase (BIONOVAS, Halifax, NS, Canada). Reverse transcription was conducted according to the manufacturer’s protocol with random primer. The synthesis condition of cDNA was set at: 65 °C for 5 min, 30 °C for 10 min, 42 °C for 60 min and 70 °C for 15 min.

### 2.3. Quantitative Real-Time PCR Analysis

Gene expression profiles were performed using the Applied BiosystemTM 7500 Real-Time PCR System (Applied Biosystems, Foster City, CA, USA) on a TOptical Thermocycler^®^ (Analytik Jena AG, Jena, Germany). The qPCR reaction volume was 20 μL in total, containing 1 μL of the cDNA template, 10 μL of the 2X qPCRBIO syGreen Master Mix, 0.8 μL each of the forward and reverse primer (10 pmol/uL) and 7.4 μL distilled water. The amplification condition was initial denaturation at 95 °C for 5 min, followed by 40 cycles of 95 °C for 5 s, 65 °C for 30 s. The melting curve and cooling were performed at the last step of qPCR. The primers used in this study are listed in Table S1. The relative expression levels of the target gene were normalized to beta-actin, a housekeeping gene and calculated by the standard 2^−ΔΔCt^ method. The changes were analyzed by unpaired sample *t*-test. Statistical significance was accepted at *p* < 0.05, and high significance was accepted at *p* < 0.01. All data were expressed as mean ± standard deviation (mean ± SD).

### 2.4. Genomic DNA Extraction and Gene Sequencing

Total genomic DNA from the intestine was extracted using the Genomic DNA Mini Kit (Geneaid) according to the manufacturer’s instructions. The genomic DNA was monitored on 1% agarose gels to analyze the DNA concentration and purity. Amplicon sequencing (16S V4: 515F-806R) was performed by using 300 bp paired-end raw reads and all the paired-end reads were assembled using FLASH v.1.2.7 [26] at Biotools, Co., Ltd. Demultiplexing was carried out based on barcode identification. As a quality control, low-quality reads (Q < 20) were discarded by the QIIME 1.9.1 pipeline [27]. If three consecutive bases were < Q 20, the read was truncated and the resulting read was retained in the data set only if it was at least 75% of the original length using split_libraries_fastq.py script in QIIME [28]. Sequences were chimera-checked using UCHIME to obtain the effective tags [29,30] and filtered from the data set; then, operational taxonomic unit (OTU) clustering at 97% sequence identity using the UPARSE [31] function in the USEARCH v.7 pipeline [32] was performed. For each representative sequence, the RDP classifier (v.2.2) algorithm [33] was employed to annotate taxonomy classification based on the information retrieved from the Silva Database v.132 [34,35]. This analysis was performed with a minimum confidence threshold of 80% for each assignment. Sequences with one occurrence (singletons) or present in only one sample were filtered out. To analyze the sequence similarities among different OTUs, multiple sequence alignment was conducted by using PyNAST software (v.1.2) [36] against the core-set dataset in the Silva database.

### 2.5. Bioinformatics and Statistical Analysis

Subsequent analysis (NCBI-Accession number: SUB7303081) of alpha and beta diversities was performed using the normalized data. Alpha diversity was indicative of the species complexity within individual samples based on the observed OTU output from the QIIME pipeline. The number of observed OTUs is the number of different species represented in the microbial community. Beta diversity parameters, the unweighted UniFrac [37,38], were calculated by using the QIIME pipeline. Normally, UniFrac is used for analyzing the comparison between two samples to achieve a UniFrac distance matrix. To further increase the group distinction, a supervised partial-least-squares discriminant analysis (PLS-DA) was used to evaluate and visualize variance based on the OTU level of the intestinal microbiota composition among the groups. PLS-DA was performed using the R package mixOmics. For statistical analysis, the significance of all species among groups at various taxonomic levels was detected using differential abundance analysis with a zero-inflated Gaussian (ZIG) log-normal model as implemented in the “fitFeatureModel” function of the Bioconductor metagenomeSeq package [39]. Moreover, Welch’s *t*-test was performed using STAMP software (v2.1.3) to identify significant differences among the groups (*p* value < 0.05) [40].

## 3. Results

### 3.1. The Intestinal Microbiota Richness and Diversity Associated with Grouper Disease

**Figure 1:**
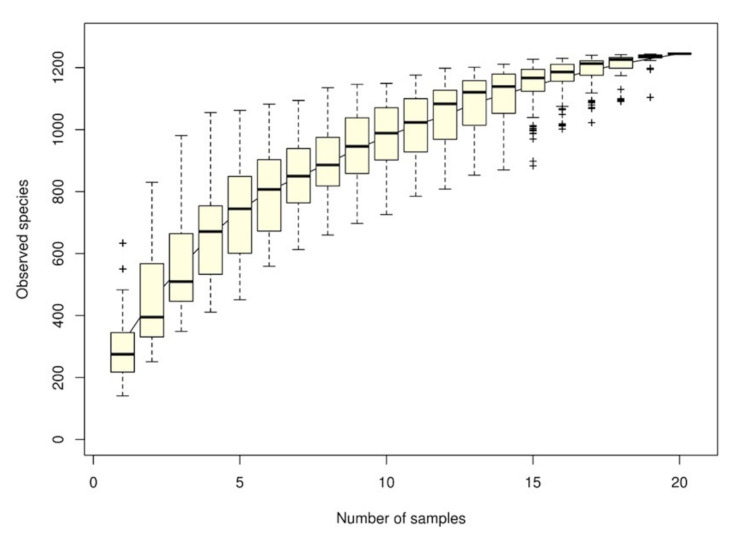
Species accumulation curve derived by grouper intestine samples and estimations of the number of intestinal microbiomes in the gastrointestinal tract of grouper. Each dot represents the total richness for all samples pooled. The x-axis is the number of samples and the y-axis is the number of observed species.

The species accumulation curve (SAC) indicated that the number of microbial OTU had a positive effect on species richness, indicating that the adequacy of sample size could generate a sample survey. When the curve became flat, the number of intestinal microbial species did not increase with the sample size. Figure 1 presents a sharp increase in the curve until a sample size of 20, indicating that the survey sample size of intestinal microbial species was adequate.

**Figure 2:**
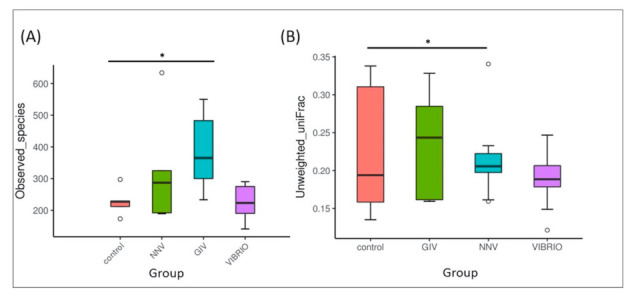
Species diversity. (**A**) Alpha diversity analysis with observed species indices was used to evaluate the number of different species in the intestinal flora. (**B**) Beta diversity analysis with unweighted UniFrac was used to evaluate the differences in microbial communities among samples in terms of species complexity [40]. The asterisk (*) represents significant difference at * *p* < 0.05; ** *p* < 0.01; *** *p* < 0.001 from controls. Note: The upper and lower range of the standard deviation are indicated in each box with a horizontal line with dots as outliers. The interquartile range is Q3-Q1 (IQ), the maximum range is Q3 + 1.5 * IQ, and the minimum range is Q1 − 1.5 * IQ, which generate the area between the minimum and maximum; a line inside the boxplot shows the median. The group names are plotted on the X-axis.

In the alpha diversity analysis, the observed species indices in Figure 2A represent differences in the species richness of control group and pathogen-infected group. The variation in the GIV group was higher than that in the other groups, the NNV and Vibrio groups, while the control group became the least abundant. Similarly, unweighted UniFrac is a qualitative beta diversity measure that compares the significant microbial differences in each sample. As shown in Figure 2B, the microbial beta-diversity was increased in the order of Vibrio, NNV, and GIV, followed by the control group. These results indicated that an immense diversity of intestinal microbiota in healthy grouper could maintain immune homeostasis in the gastrointestinal tract.

### 3.2. The Intestinal Flora Community Composition of Uninfected and Diseased Grouper

**Figure 3:**
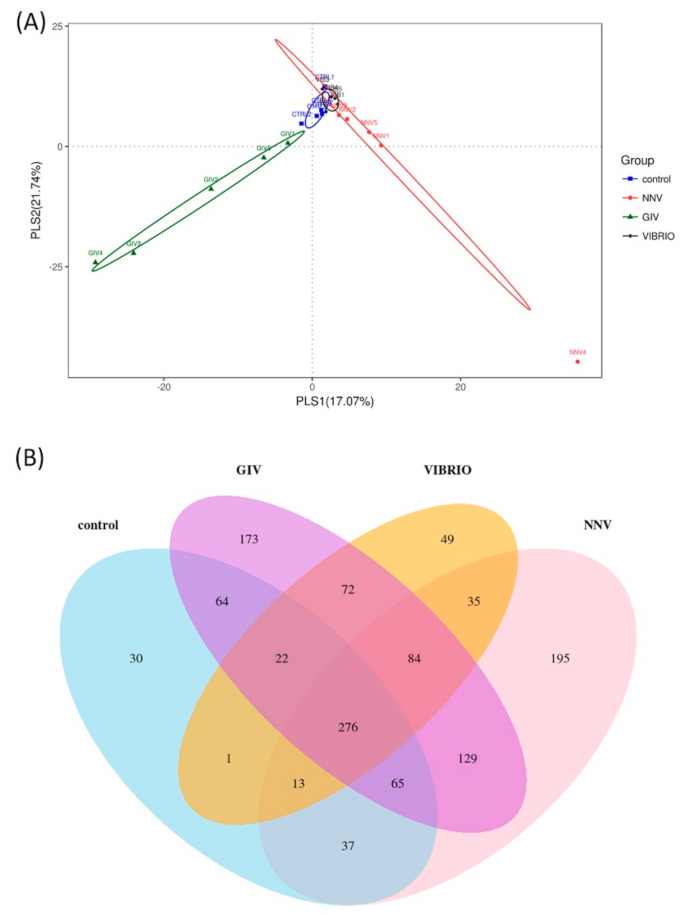
Microbial community composition. (**A**) Partial least-square-discriminant analysis (PLS-DA) of sample distribution (blue squares represent the control, red circles represent NNV, green triangles represent GIV and black dots represent Vibrio). (**B**) Venn diagram representing OTU richness and overlap of microbial communities in the control, GIV, Vibrio and NNV groups (red), which are represented by blue, purple, yellow and red color, respectively. The total OTU richness was 6372 OTUs among the four groups.

Based on PLS discriminant analysis (PLSDA), each dot represents a sample. The PLSDA diagram of the distribution of microbes significantly separated the GIV group and the other groups, is shown in Figure 3A. Similar distributions were found in the control and Vibrio groups. The NNV group was correlated with these two groups. These results provided a good dataset for clearly understanding the variability and correlation in control group and pathogen-infected groups. A Venn diagram shows the different relationships among the control, NNV, GIV and Vibrio groups (Figure 3B). In total, 1245 OTUs were found in these four groups. There were 276 OTUs in the overlapping area; these OTUs were common to all groups. Furthermore, the nonoverlapping region revealed the unique OTUs in each group, including 30 OTUs, 173 OTUs, 49 OTUs and 195 OTUs that belonged to the control, GIV, Vibrio and NNV groups, respectively. Sixty-four OTUs were shared between the control and GIV groups; one OTU was shared between the control and Vibrio groups; and 37 OTUs were shared between the control and NNV groups. A small number of OTUs were shared between the NNV, GIV, Vibrio groups and the control group, and the overall variation of intestinal microbial communities in pathogen-infected grouper seemed distinct from that of uninfected grouper.

### 3.3. Interaction between Disease and Intestinal Microbiomes

**Figure 4:**
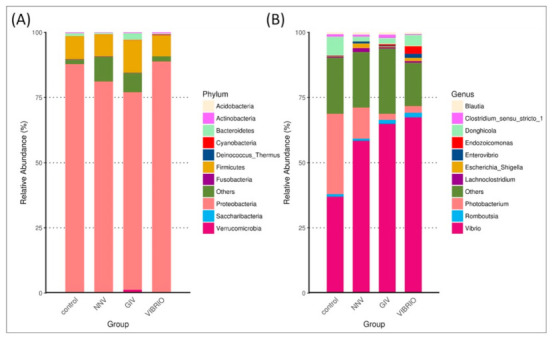
Species abundance distribution displayed the top ten most abundant at the (**A**) phylum and (**B**) genus levels. The distribution was visualized as microbiomes with different relative abundances at classification levels and their relative abundance proportions. Typically, the data intuitively distinguished the differences in the abundance of bacteria between healthy and diseased grouper. The X-axis shows the group names; the Y-axis shows the percentage of relative abundance.

We determined the top ten taxonomic classifications to examine the relative abundance and proportions of microbial phyla (Figure 4A) and genera (Figure 4B) in uninfected and diseased grouper. 

**Figure 5:**
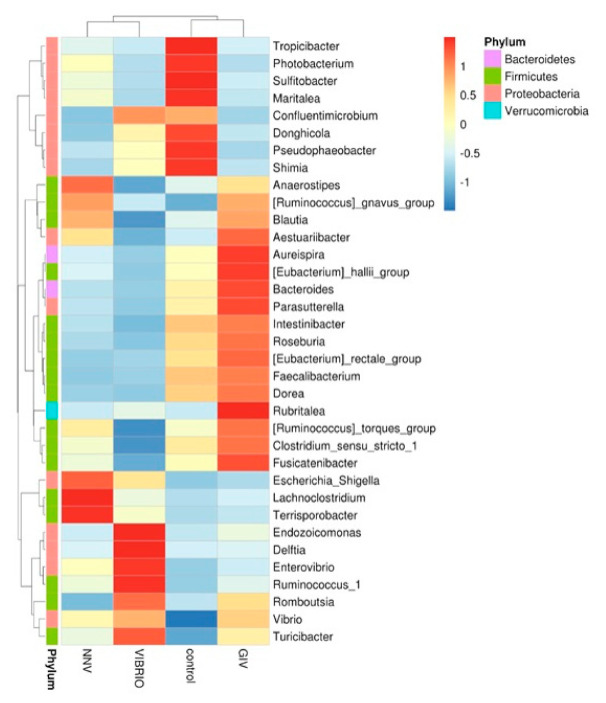
Bacterial abundance heatmap at the genus level was established for the control, nervous necrosis virus (NNV), grouper iridovirus (GIV) and Vibrio groups. The blue color indicates that the abundance level was less than the mean level, while the red color indicates that the abundance level was higher than the mean level.

The intestinal microbiome (at the phylum and genus level) showed different microbial communities in each treatment group. Intestinal microbial communities in each group were dominated by two bacterial phyla: *Proteobacteria* and *Firmicutes,* with control (88% and 10%), NNV group (81% and 9%), GIV group (76% and 13%) and Vibrio group (89% and 8%), respectively. In the control group, the most abundant genus level was *Vibrio* (36%), followed by *Photobacterium* and *Donghicola* (30% and 7%, respectively). On the other hand, the genus *Vibrio* had higher connectivity with the NNV, GIV and Vibrio-infected groups, indicating that both viral and bacterial diseases affect the communities of harmful microorganisms. *Endozoicomonas* accounted for 2% and was overrepresented in the Vibrio group, while the proportion of *Escherichia Shigella* in the NNV and Vibrio groups (1%) was greater than that in the control and GIV groups, accounting for 0.2% and 0.3%, respectively. Compared with the NNV (1%) and GIV groups (2%), *Donghicola* was abundant in the control (7%) and Vibrio groups (4%). The results of taxonomic classification (Figure 5) revealed that *Proteobacteria* might play a key role in both heathy and pathogen-infected grouper. Thus, we further analyzed the top 35 genera and generated a taxa heatmap to analyze the microbiome annotation and abundance information. The microbial communities of the control group consisted of *Tropicibacter, Photobacterium, Sulfidobacter, Maritalea, Donghicola, Pseudophaebacter* and *Shimia*. *Lachnoclostridium* and *Terrisporobacter* were most abundant genera in NNV-infected grouper. Four genera were enriched in the Vibrio group: *Endozoicomonas, Delftia, Enterovibrio* and *Ruminococcus*. In addition, *Aureispira, Eubacterium hallii, Bacteroides, Parasutterella, Rubritalea* and *Fusicatenibacter* were highly abundant in the GIV group.

### 3.4. Differential Abundance Analysis and Statistical Analysis

**Figure 6:**
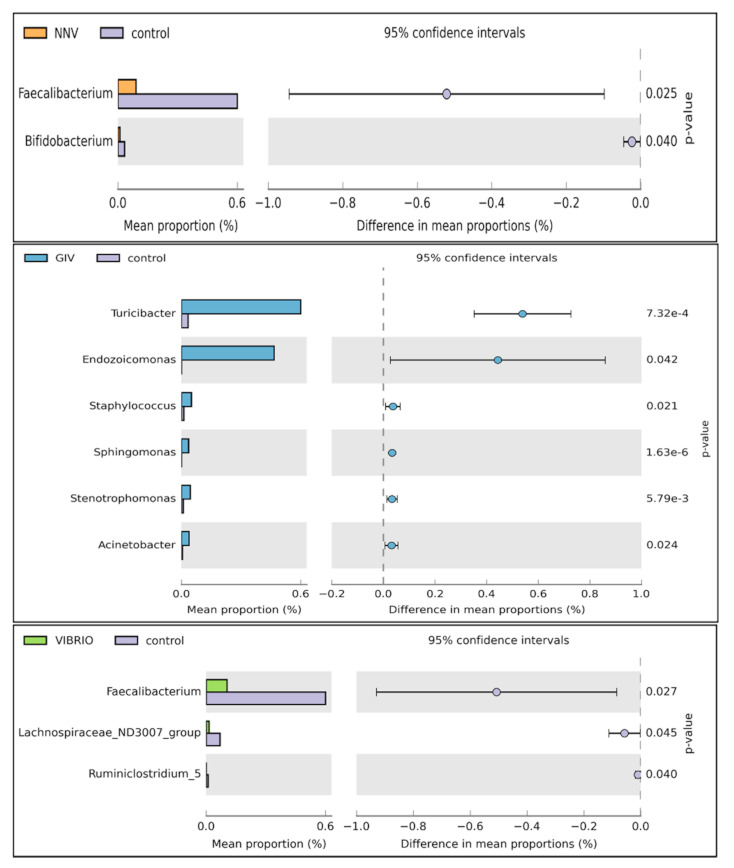
Comparison of uninfected and different disease grouper intestinal microbiota to characterize differential abundances between the two groups. Note: The left chart shows the mean abundance level and ratio between two groups, while the right chart shows the 95% confidence intervals. The probability value (*p* value < 0.05) is plotted on the right-hand side.

**Figure 7:**
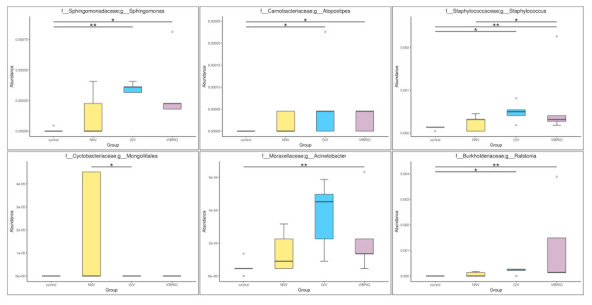
MetagenomeSeq annotated the species with significant differences (* *p* value < 0.05; ** *p* value < 0.01) among the groups to conduct an intensive study of community structure differences. Top row, genus *Sphingomonas*, *Atopostipes*, *Staphylococcus*; Bottom row, genus *Mongoliitalea*, *Acinetobacter* and *Ralstonia*.

Welch’s *t*-test was performed on different pathogen-infected groups to further study the microbial community structure. Through statistical analysis, the microbiomes with significant difference in abundance between groups could be found depending on the OTU abundance features. As shown in Figure 6, the induction of *Faecalibacterium* and *Bifidobacterium* was significantly affected in the control group compared to the NNV group. Increases in *Endozoicomonas*, *Staphylococcus, Sphingomonas, Stenotrophomonas and Acinetobacter* were observed in the GIV group. In the Vibrio and control groups, *Faecalibacterium* was more abundant and was clearly observed in healthy grouper. From these results, we found that the pathogen may drive changes in the abundance of microbiomes. To support this view, we further investigated the relationship between the intestinal microbiomes of uninfected grouper and NNV, GIV and Vibrio-infected grouper. Therefore, we tried to clearly understand the potential roles of healthy and diseased grouper, and we subsequently assayed the differentially abundant species by metagenomeSeq in the control, NNV, GIV and Vibrio groups (Figure 7).

Compared with control group, NNV, GIV and Vibrio groups showed an increase in the abundances of genus *Sphingomonas*, *Atopostipes*, *Staphylococcus* and *Acinetobacter.* Among these, *Sphingomonas* spp. and *Staphylococcus* spp., which are known to be harmful bacteria in both human and aquatic animals. In addition, *Mongoliitalea* spp. was clearly superior to the NNV group, whereas the vibrio group was enriched with *Ralstonia* spp. In conclusion, there was a significant difference among uninfected, NNV-infected, GIV-infected and Vibrio-infected microbiota, which implied that the normal and healthy composition of grouper might be altered through viral and bacterial infection.

### 3.5. Pathogenic Infection Up-Regulates the Pro-Inflammatory Cytokine Gene Expression in E. coioides Intestine and Head Kidney

**Figure 8:**
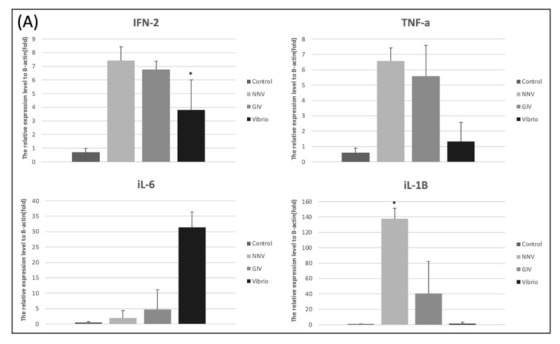

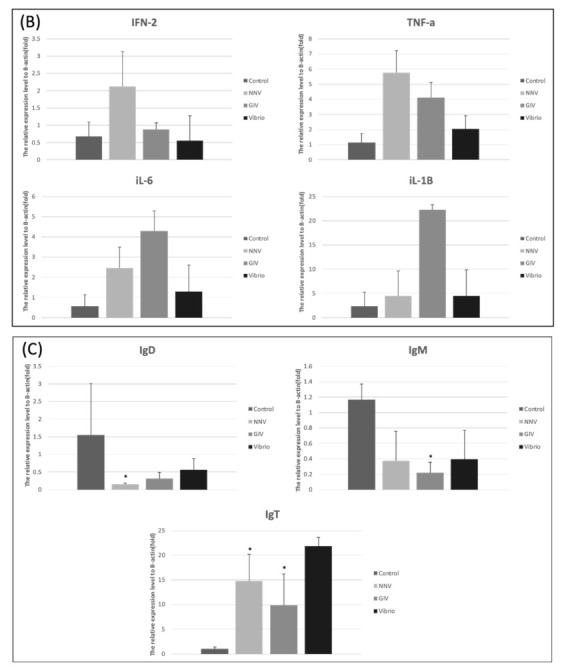
Innate immune-related genes expression in the intestine and head kidney of *E. coioides* after infection with NNV, GIV and *V. harveyi*. (**A**) The expression levels of IFN-2, TNF-α, iL-6 and iL-1β in intestines. (**B**) The expression levels of IFN-2, TNF-α, iL-6 and iL-1β in head kidney. Adaptive immune-related genes expression in the intestine of *E. coioides* after infection with NNV, GIV and *V. harveyi*. (**C**)The different Ig isotopes (IgD, IgM and IgT) expression in the intestines of control group and pathogen infected (NNV, GIV and Vibrio group) group. The relative mRNA levels were normalized by β-actin. Data presented are presented as mean ± SD (n = 5) and the asterisk (*) represented significant difference at * *p* < 0.05; ** *p* < 0.01; *** *p* < 0.001 from controls.

As shown in Figure 8A, treatment with NNV, GIV and Vibrio caused an increase of proinflammatory response in intestines. The expression level of IFN-2 and TNF-*α* were significantly increased in the Vibrio group. Similarly, iL-6 and iL-1*β* expression levels in Vibrio group were higher than control group. On the other hand, the higher induction TNF-*α*, iL-6 and iL-1*β* were observed in NNV and GIV group compared with the control group. In head kidney, we found the IFN-2 and TNF-*α* highly expressed in NNV group. iL-6 and iL-1*β* expressions in the GIV and Vibrio groups were higher than control group. Our results showed the innate immune genes were upregulated in both intestine and head kidney after pathogen infection. To evaluate whether the immune-related genes were expressed in *E. coioides* after pathogen infection, we analyzed the immunoglobulins (antibodies) in the intestines (Figure 8C). The expression of IgD and IgM was the most upregulated in the control group compared to pathogen infected group, whereas no significant changes were shown in IgT expression. In contrast, we found that IgT expression of the pathogen infected group increased when compared to the control group. 

## 4. Discussion

Recent studies have suggested that microbiome profiling has the capacity to become a feasible application for screening candidate microbiomes against animal or plant diseases, including colorectal cancer and irritable bowel disease in humans, leaf spot disease in plants, and bleaching disease in aquatic animals [41,42,43,44]. Based on our case study, we used metagenomics analysis to analyze the function of the microbiome and the interaction between the microbiome and host. Indeed, this tool can help identify potential therapeutics [45,46]. 

We aimed to understand the differences in the intestinal microbiota of *Epinephelus coioides* under normal conditions and pathogen pressure. In this study, we investigated the composition of intestinal microbiomes from uninfected individuals and NNV, GIV and *V. harveyi*-infected individuals. NNV usually affects the nervous system, causing behavioral abnormalities and extremely high mortalities [25,47], whereas iridovirus infection leads to high mortality in many economic marine and freshwater fish, especially GIV-infected grouper, the mortality of which ranges from 30% to 100% [48,49,50]. In addition, *V. harveyi* is an opportunistic pathogen causing skin injury and muscle necrosis disease in grouper [51]. As stated above, grouper is susceptible to these infectious diseases and causes significant economic losses in aquaculture [52]. We further examined whether these diseases could alter the composition of the intestinal flora. In Figure 2A, we assumed that the increase in species richness in the intestinal microbiota, during grouper infection with both virus and bacterial pathogens, might help to enhance resistance against the pathogens. Figure 2B indicates that an immense diversity of intestinal microbiota in healthy grouper could maintain immune homeostasis in the gastrointestinal tract. Our findings, which indicated no significant differences in intestinal microbiome diversity (data not shown), are consistent with a previous report [53]. Apart from that, PLSDA (Figure 3A) presented the intestinal microbiome communities from uninfected and infected grouper. The GIV group clustered distinctly among all groups. The NNV and Vibrio groups remained close to the control group, indicating that GIV might affect the enrichment and diversity of intestine communities. Previous studies revealed that the environment, dietary habits or disease also influence the structure of the intestinal bacteria in aquatic animals [18,22,54,55].

The intestinal communities of a majority of marine fish harbor a higher proportion of *Proteobacteria* and *Firmicutes* [56,57,58]. In our study, these two phyla were enriched in all groups, revealing that they are the most common bacteria in the intestinal flora of marine fish [59]. At the genus level, the intestine communities of the control group were dominated by *Tropicibacter, Photobacterium, Sulfidobacter, Maritalea, Donghicola, Pseudophaeobacter* and *Shimia*. Among these, many *Sulfitobacter* strains, which are considered sulfite oxidizers, have the ability to produce sulfite oxidases, including the oxidation of organic and inorganic sulfur compounds and carbon monoxide, which can be used in biosensor systems for detecting sulfite [60,61,62,63,64]. Researchers from Iran discovered that *Maritalea* is a radiation-resistant microorganism that acts as a remoistening agent to endure extreme dryness [65]. *Phaeobacter* spp., such as *P. inhibens* and *P. gallaeciensis*, have been used as probiotics against *Vibrio* pathogens in scallop and algae cultures [66,67]. *Tropicibacter, Photobacterium, Donghicola* and *Shimia* are genera that are abundant in marine organisms or seawater, and *Photobacterium* mainly live in symbiotic relationships with aquatic organisms [68,69,70,71]. Additionally, we detected an abundance of *Lachnoclostridium* and *Terrisporobacter* in the NNV group. Previous studies identified that Lachnoclostridium is related to several viral diseases and has high potential to become a bacterial marker for colorectal adenoma detection [72,73]. In addition, *Terrisporobacter* was formerly known as a pathogen in humans [74]. The genera *Enterovibrio, Delftia, Endozoicomonas* and *Ruminococcus* were abundant in the Vibrio group; these genera are opportunistic bacterial pathogens in both humans and fish [75,76,77,78,79,80]. After infection with GIV, the intestinal microbiota was associated with a wide range of *Aureispira, Eubacterium hallii, Bacteroides, Parasutterella, Rubritalea* and *Fusicatenibacter*. *Bacteroides* and *Parasutterella* often act as virulence factors in the intestine to form intestinal chronic inflammation [81,82]. Nonetheless, *Eubacterium hallii* has the ability to maintain intestine metabolic balance due to the formation of propionate [83]. In short, the relationships between these commensal bacteria and their hosts remain unknown, and they may play an important role in the intestinal immune system of grouper, suggesting that the key bacteria present in each group could be further validated as screening microbiome markers for NNV, GIV and *V. harveyi* infection.

In order to better understand the differential abundance of significantly different microbiomes, we explored the expression profile of microbiomes via Welch’s *t*-test. The upregulation of *Faecalibacterium* and *Bifidobacterium* in control group is consistent with the results of previous microbiota studies [84,85,86,87,88]. Similar studies have shown that *Faecalibacterium* is predominantly expressed in healthy individuals [89]. These results demonstrated that these two microbiomes could be novel complex supplements considered for use in aquaculture. In contrast, the infection group had an increased abundance of pathogenic bacteria. For example, *Staphylococcus warneri* was generally recognized as a fish pathogen [90] and is the major cause of infectious diseases in spotted rose snapper [91] and rainbow trout [92,93]. In MetagenomeSeq analysis, we found *Mongoliitalea* spp. was clearly superior to the NNV group, whereas the vibrio group was enriched with *Ralstonia* spp., some species reported as infectious bacteria on human, which cause meningitis, respiratory infection and catheter related infection [94]. Acinetobacter spp., which are abundant in NNV, GIV and Vibrio-infected grouper, were identified as serious pathogens that could lead to major disease outbreak in *Labeo catla* [95]. In the Vibrio group, *Ralstonia* spp. was a common bacterium isolated from diseased fish [96]. This analysis showed that grouper have a weak immune system that may allow some harmful bacteria to colonize the intestinal tract.

Fish immunity consists of two components, innate and adaptive immune system [97]. Recent studies revealed that cytokines play appreciable innate immune factors in fish which displayed as antibacterial, antifungal, antiviral and antiprotozoal agents [98]. The gut-associated lymphoid tissues (GALT) contain a variety of immune cells and play a critical role in maintaining the balance between pathogenic and commensal microbiota [99]. Moreover, the microbiota has a positive effect on immune regulatory functions of the intestine by stimulating the immune response during pathogen infection [100]. Valero Y et al., [101] noticed that antibacterial and antiviral activity were modulated by several innate and adaptive immune genes. We analyzed different genes, including proinflammatory cytokines and immunoglobulins, to determine the innate and adaptive immune responses triggered by viral and bacterial infections. Previous studies reported that different proinflammatory cytokines production was induced by the pathogens and host commensal communities [102,103,104]. Similarly, all the expression of proinflammatory cytokines (IFN-2, TNF-*α*, iL-6 and iL-1*β*) showed upregulation in the intestines and head kidneys of pathogen-infected groupers compared with the control group. As expected, the immunoglobulin-based adaptive immune system (Figure 8B) showed high expression of IgT in intestines of pathogen-infected grouper, whereas IgM and IgD in intestines showed no substantial difference between uninfected and pathogen-infected grouper. This result revealed that immunoglobulin T presented a specialized function in the intestinal mucosa immunity after infection with viral and bacterial pathogens. Strong evidence from a USA team indicated IgT has a specialized function in mucosal immunity, acting as mammalian IgA, which plays a critical role during viral and bacterial pathogen infection in teleost [105].

In summary, we examined the intestinal microbiota profile of uninfected *E. coioides* and *E. coioides* infected with different types of pathogens. Our results clearly indicated that disease dramatically affected the diversity and composition of the fish intestinal microbiota. Interestingly, we discovered that the intestinal flora of diseased grouper contained a high abundance of harmful bacteria. In contrast, beneficial bacteria were overrepresented in the control group. However, the symbiotic relationships between either bacteria and bacteria or bacteria and host are currently unknown, and they might be key to helping the host against both viral and bacterial disease. Thus, we speculated that infectious diseases are associated with the intestine immune system, which eventually gives rise to the proinflammatory response [106]. Perhaps a better understanding of the roles these bacteria play may allow us to develop a novel dietary strategy, such as multistrain probiotics, and identify novel bacterial markers for the diagnosis of NNV, GIV and *V. harveyi* infections. Additionally, identification of the composition and function of disease-associated microorganisms in inflammatory and immune response can be further investigated and considered for the prevention and control of infectious diseases in the aquaculture industry.

## Supplementary Materials

The following are available online at https://www.mdpi.com/2075-1729/11/2/99/s1, Table S1: Sequence of primers used in this study.
