# Supplementary material for: The Alteration of Intestinal Microbiota Profile and Immune Response in Epinephelus coioides during Pathogen Infection
Source: Life (Basel). 2021 Jan 28;11(2):99. doi: 10.3390/life11020099 (PMC7912457; doi:10.3390/life11020099)
Supplement: Supplementary file 1 [file life-11-00099-s001.pdf]

life

Supplementary Materials

The Alteration of Intestinal Microbiota Profile and Immune Response in *Epinephelus coioides* during Pathogen Infection

Table S1. Sequence of primers used in this study.

Primer Name	Nucleotide Sequences (5′-3′)	Primer Usage
β-actin-F	TCCACCgCAAATgCTTCTAA	real-time PCR
β-actin-R	TgCgCCTgAgTgTgTATgA	real-time PCR
IgD-F	ATTTTgACgCCAAgTTgACC	real-time PCR
IgD-R	TgCCAGCTTGAAAATGATG	real-time PCR
IgM-F	CTATCTGCTGGGCAGGTgTT	real-time PCR
IgM-R	GCAGCAGAATCTTCAGTCTTCA	real-time PCR
IgT-F	TGTGTCAAAGTCTGCCTGGGATTCA	real-time PCR
IgT-R	CTTAGGAGGTGGAGGAGGCTTTTG	real-time PCR
IFN-2-F	TGGAGGCGTACGAAAAGCTG	real-time PCR
IFN-2-R	GCTCGTTGTACCGGTGTTTC	real-time PCR
TNF-α-F	GCAAAGCCTCGCTGATG	real-time PCR
TNF-α-R	GCCCAGATAAATGGCGTTGT	real-time PCR
IL-6-F	GGAGAGGCTCAGAGGAAG	real-time PCR
IL-6-R	ACACCTGAGTGTGAGAACAGTAA	real-time PCR
IL-1β-F	CCAGCGTTGAGGGCAGAA	real-time PCR
IL-1β-R	ATCGTCTCCAGATGTAAGGTT	real-time PCR
